# *Cordyceps cicadae* polysaccharides alleviate hyperglycemia by regulating gut microbiota and its mmetabolites in high-fat diet/streptozocin-induced diabetic mice

**DOI:** 10.3389/fnut.2023.1203430

**Published:** 2023-08-03

**Authors:** Yanan Wang, Zaizhong Ni, Jinting Li, Ying Shao, Yidan Yong, Wendi Lv, Simeng Zhang, Tingwei Fu, Anhui Chen

**Affiliations:** College of Food and Bioengineering, Xuzhou University of Technology, Xuzhou, Jiangsu, China

**Keywords:** *Cordyceps cicadae*, polysaccharides, hypoglycemic, gut microbiota, metabolites

## Abstract

**Introduction:**

The polysaccharides found in *Cordyceps cicadae* (*C. cicadae*) have received increasing academic attention owing to their wide variety of therapeutic activities.

**Methods:**

This study evaluated the hypoglycemic, antioxidant, and anti-inflammatory effects of polysaccharides from *C. cicadae* (CH-P). In addition, 16s rDNA sequencing and untargeted metabolomics analysis by liquid chromatography-mass spectrometry (LC-MS) were used to estimate the changes and regulatory relationships between gut microbiota and its metabolites. The fecal microbiota transplantation (FMT) was used to verify the therapeutic effects of microbial remodeling.

**Results:**

The results showed that CH-P treatment displayed hypoglycemic, antioxidant, and anti-inflammatory effects and alleviated tissue damage induced by diabetes. The CH-P treatment significantly reduced the *Firmicutes/Bacteroidetes* ratio and increased the abundance of *Bacteroides, Odoribacter, Alloprevotella, Parabacteroides, Mucispirillum,* and significantly decreased the abundance of *Helicobacter* and *Lactobacillus* compared to the diabetic group. The alterations in the metabolic pathways were mostly related to amino acid biosynthesis and metabolic pathways (particularly those involving tryptophan) according to the Kyoto Encyclopedia of Genes and Genomes (KEGG) pathway enrichment analysis. Correlation analysis showed that *Bacteroides, Odoribacter, Alloprevotella, Parabacteroides,* and *Mucispirillum* were positively correlated with indole and its derivatives, such as 5-hydroxyindole-3-acetic acid. Indole intervention significantly improved hyperglycemic symptoms and insulin sensitivity, and increased the secretion of glucagon-like peptide-1 (GLP-1) in diabetic mice. FMT reduced blood glucose levels, improved glucose tolerance, and increased insulin sensitivity in diabetic mice. However, FMT did not significantly improve GLP-1 levels.

**Discussion:**

This indicates that *C. cicadae* polysaccharides alleviate hyperglycemia by regulating the production of metabolites other than indole and its derivatives by gut microbiota. This study provides an important reference for the development of novel natural products.

## 1. Introduction

Type 2 diabetes mellitus (T2DM) is a metabolic disease characterized by insulin resistance and relative insulin insufficiency, and it is one of the most prevalent worldwide public health challenges (1). The number of T2DM and T2DM-related complications continues to increase, despite great efforts to combat the disease. Currently, many drugs have been used to treat T2DM, including sulfonylureas, biguanides, α-glucosidase inhibitors, and others. However, there are adverse effects associated with these drugs, including hypoglycemia, liver damage, gastrointestinal symptoms, and weight gain. Therefore, there is an urgent need to develop more effective and safer drugs to treat T2DM.

Many natural products are considered acceptable and alternative therapeutic options for treating diabetes and its related complications because of their efficacy, multiple beneficial effects, and minimal toxicity or side effects (2). *Cordyceps cicadae* (*C. cicadae*) is an intriguing entomogenous fungus that belongs to the *Claviciptaceae* family. It parasitizes on the larvae of cicada and has been used as a tonic food and herbal medicine to treat palpitations, infantile convulsions, chronic kidney diseases, heart palpitations, dizziness, and eye disease for hundreds of years (3, 4). Futhermore, *C. cicadae* has multiple effects, such as antioxidant, anti-inflammatory, antitumor effects, and blood glucose regulation (5, 6). Polysaccharides are one of the major bioactive ingredients found in *C. cicadae* that have received increasing academic attention owing to the wide variety of therapeutic activities (7). Crude polysaccharides from *C. cicadae* significant reduced the hyperglycemia and improved the hyperlipidemia of diabetic rats induced by alloxan monohydrate (8). *Cordyceps cicadae* polysaccharides also improved insulin resistance and glucose tolerance in rats with diabetic nephropathy (9). Therefore, *C. cicadae* polysaccharides may serve as potential drugs to prevent and treat T2DM. However, the underlying mechanisms of *C. cicadae* polysaccharides in hyperglycemia and diabetes requires further exploration.

An increasing number of studies have shown that polysaccharides can exert therapeutic effects in the treatment of obesity and diabetes by regulating intestinal microbial disorders. High-molecular-weight polysaccharides from *Ganoderma lucidum* reduce body weight, inflammation, insulin resistance, and reverse gut dysbiosis in high-fat diet (HFD)-induced obese mice (10). Polysaccharides from *Hirsutella sinensis* reduce obesity, inflammation, and diabetes-related symptoms by modulating the gut microbiota composition (11). Apple polysaccharides exert health benefits by inhibiting gut dysbiosis and chronic inflammation, and modulating gut permeability in HFD-induced dysbiosis rats (12). Monosaccharides and short-chain fatty acids (SCFAs) that are beneficial for host and intestinal health by modulating obesity, diabetes, and other metabolic diseases are produced by the hydrolysis and fermentation of polysaccharides by intestinal microorganisms (13). Nevertheless, the beneficial effects of *C. cicadae* polysaccharides on gut microbiota dysbiosis and their mechanisms of improvement in diabetes are not fully clarified. This study investigated the hypoglycemic, antioxidant, and anti-inflammatory effects of *C. cicadae* polysaccharides. The abundance and changes in the gut microbiota were examined using 16S rDNA gene sequencing. Nontargeted metabolite analysis was conducted using a liquid chromatography-mass spectrometry (LC–MS) platform (Waters, UPLC; Thermo, Q Exactive). This study provides an important reference to develop novel fungal polysaccharide drugs to prevent diabetes and its associated complications.

## 2. Materials and methods

### 2.1. Polysaccharides preparation from *Cordyceps cicadae*

Hot water extraction and ethanol precipitation were used to extract crude polysaccharides from *C. cicadae*, which stored in our laboratory. Briefly, dried *C. cicadae* powder was dissolved in distilled water according to the solid–liquid ratio at 1:30 and was reflux extracted at 90°C for 3 h. The extract was collected after filtration with the 0.22 μm filter paper (Whatman, China) and concentrated using a rotary evaporator (SENCO, China) at 65°C under a vacuum. The concentrate was precipitated by adding anhydrous ethanol at a ratio of 1:4 (v/v) and incubated overnight at 4°C. Precipitates were obtained by centrifugation at 5,000 × *g* for 10 min at 4°C and dissolved in distilled water. The pigments in the crude polysaccharides were decolorized using activated carbon in a water bath at 60°C for 2 h, and the proteins were removed using the Sevag method. The solution was dialyzed in dialysis bag (3,500 Da *m_w_* cut-off) for 48 h at 4°C using distilled water, and the crude polysaccharides (CH-P) were obtained after freeze-drying. Since the polysaccharides characterization of *C. cicadae*, including chemical composition, molecular weight, and characteristic groups, were detail described in many studies (9, 14–16), the hypoglycemic activity of crude polysaccharides by regulating gut microbiota and its metabolites was investigated in this study.

### 2.2. Animals treatment

Male SPF grade C57/BL6J mice (20.0 ± 2.0 g) were purchased from Pengyue Experimental Animal Breeding Co., Ltd. (Jinan, China). A mouse diabetes model was established as described in our previous study (17). Briefly, the mice were acclimatized for 1 week under a 12-h light/dark cycle at 23 ± 2°C and a relative humidity level of 50 ± 5%. The mice had access to food and water *ad libitum*. The mice were randomly divided into normal control (Con, *n* = 5) and diabetic (*n* = 20) groups. The Con group was fed a maintenance diet throughout the experiment. The diabetic group (*n* = 20) was fed a high-fat diet (66.5% maintenance diet, 10% lard, 20% sucrose, 2.5% cholesterol, and 1% sodium cholate) for 4 weeks, followed by a single intraperitoneal injection of 100 mg/kg streptozotocin (STZ, sigma-aldrich, United States) after fasting overnight. The Con group was injected with an equal volume of citrate buffer (0.1 mol/L, pH 4.4). Mice with fasting blood glucose levels higher than 11.1 mmol/L 1 week after STZ injection were considered diabetic.

Diabetic mice were randomly divided into five groups: (1) diabetic control group (Dia, *n* = 5) treated with saline; (2) metformin positive group (Met, *n* = 5) treated with metformin at dose of 300 mg/kg; and (3) diabetic group were orally administered CH-P at doses of 200 mg/kg (CH-P-200), 400 mg/kg (CH-P-400), and 800 mg/kg (CH-P-800) body weight for 3 weeks. All animals were treated once a day. Food intake, water intake, and body weight were measured daily, and blood glucose levels were measured once a week throughout the study. The oral glucose tolerance test (OGTT) was performed as previously described in our study (18).

A homeostatic model assessment was used to assess the changes in insulin sensitivity (HOMA-IS). The area under the curve (AUC) and HOMA-IS were calculated using the following formulas:


(1) $$\begin{array}{l} \mathrm{AUC}\;\left(\frac{\min.\mathrm{mmol}}{L}\right)=\frac{1}{2}\times \left[\mathrm{BG}\;\left(0\min\right)+\mathrm{BG}\;\left(120\min\right)\right]\\ \;\;\;\;\;+\left[\mathrm{BG}\;\left(90\min\right)+\mathrm{BG}\;\left(60\min\right)-\mathrm{BG}\;\left(30\min\right)\right]\times 30\min\;\; \end{array}$$

(2) HOMA−IS=1/(FBG∗FINS)

The mice were sacrificed at the end of the experiment and blood and tissues samples were collected for further analysis.

### 2.3. Biochemical parameters analysis and histological analysis

Blood samples were incubated for 30 min at 37°C, and then centrifuged at 3,000 × *g* for 15 min at 4°C to obtain serum for biochemical analysis. Commercially available kits were used to measure lipid indicators (total cholesterol (TC), triglycerides (TG), high-density lipoprotein cholesterol (HDL-C), and low-density lipoprotein cholesterol (LDL-C)); antioxidant parameters (malondialdehyde (MDA) and total superoxide dismutase (T-SOD) activity); and inflammatory factors (TNF-α, IL-6, and IL-1β) according to the manufacturer’s instructions.

Tissues from pancreas, liver, heart, kidney, adipose, and aorta were fixed using 4% paraformaldehyde (biosharp, China), then dehydrated in a graded series of ethanol (70, 80, 90, 95, and 100%) and embedded in paraffin. The tissues were sectioned to a thickness of 5 μm using a slicer (Leica, German) for staining with hematoxylin–eosin (H&E).

### 2.4. Gut microbiota analysis

Total DNA was extracted from fecal samples using an AxyPrep DNA recovery kit (Axygen, United States) according to the manufacturer’s instructions. The quality of the extracted DNA was determined by 1% agarose gel electrophoresis, and the concentration and purity of DNA was determined using a NanoDrop 2000 spectrophotometer (Thermo Fisher Scientific, United States). The V3-V4 variable region of the 16S rDNA gene was amplified by PCR using the following primers: 357F (5′-ACTCCTACGGRAGGC AGCAG-3′) and 806 R (5′-GGACTACHVGGGTWTCTAAT-3′). The amplicon was sequenced using the NGS Illumina (Illumina, United States) platform and used to construct sequencing libraries. All sequences were divided into operational taxonomic units (OTUs) based on their similarity levels, and the OTUs were statistically analyzed at a similarity level of 97%. The representative OTU sequence was compared with the database for species annotation using Mothur (classify.seqs) software. Alpha diversity was estimated using the Chao and Shannon indices to evaluate species richness and species diversity of the samples. β diversity was evaluated by principal coordinates analysis-based OTU (PCoA) developed by Jaccard and Bray-Curtis to reveal the aggregation and dispersion of samples. Linear discriminant analysis Effect Size (LEfSe) was performed to generate a cladogram to identify different biomarkers in the gut microbiota [linear discriminant analysis (LDA) > 3, *p* < 0.05]. The metabolic pathways involved in the changes of the metabolites were determined using KEGG pathway enrichment analysis.

### 2.5. Untargeted metabolomics analysis by LC-MS

50 mg fecal samples were weighed and fully mixed with 800 μL 80% methanol. The samples were grinded with high-throughput tissue grinder (SCIENTZ-48, China) at 65 Hz for 180 s, and ultrasound-treated (PS-80A, China) at 4°C for 30 min. The samples were then incubated at −40°C for 1 h, vortexed for 30 s, and centrifuged at 12,000 × *g* for 15 min at 4°C to collect the supernatant. Two hundred microliters supernatant was mixed with 5 μL dichlorophenylalanine (0.14 mg/mL, aladdin, China) for further analysis. Untargeted metabolomics was analyzed using an LC–MS platform (Waters, UPLC; Thermo Fisher Scientific, Q Exactive). Mobile phase A was 0.05% v/v formic acid (aladdin, China) and mobile phase B was acetonitrile (sigma-aldrich, United States). The injection volume was 5 μL and the automatic injector temperature was 4°C. The ACQUITY HSS T3 column (2.1 mm × 100 mm, 1.8 μm, Waters) temperature was set to 40°C at a flow rate of 0.300 mL/min. Data were monitored in positive ion mode (POS) and negative ion mode (NEG). Quality control (QC) samples were prepared by mixing equal amounts of fecal samples to be tested. All original LC–MS data were imported into Compound Discoverer 3.1 data processing software to conduct spectrum processing and database searching to obtain the qualitative and quantitative results of the metabolites. Multivariate statistical analyses [including principal component analysis (PCA) and partial least squares discriminant analysis (PLS-DA)] were used to reveal differences in metabolites between the different groups. Hierarchical clustering and metabolite correlation analyses were used to reveal the relationships between samples and metabolites. The biological significance of the metabolites was explained through functional analysis of the metabolic pathways. Hierarchical clustering analysis was used to interpret the correlation between gut microorganisms and metabolites.

### 2.6. Indole intervention and fecal microbiota transplantation

Fecal samples were collected from the C-P-800 group into sterile frozen pipes using a sterile toothpick, quickly snap-frozen in liquid nitrogen, and stored at −80°C until further processing. 300 mg fresh fecal pellets were dissolved and suspended in 2 mL of normal saline. Homogenized fecal mixtures were centrifuged at 2,000 × *g* for 1 min at 25°C, and the supernatants were transferred to new tubes that were immediately administered to the mice by oral gavage. The diabetic mice were divided into a diabetic control group (F-DG, *n* = 5), an indole intervention group (I-TG, *n* = 5), and an FMT treatment group (F-TG, *n* = 5). Healthy C57/BL6J mice were randomly selected for the normal control group (F-NG; *n* = 5). Mice in the F-TG group were treated with 300 μL fecal suspensions collected from CH-P treatment groups and the indole intervention group was treated with a dose of 50 mg/kg by oral gavage daily for 4 weeks. F-NG and F-DG mice received equivalent volumes of normal saline.

### 2.7. Statistical analysis

Data are expressed as mean ± standard deviation (SD). Statistical analyses were performed using GraphPad Prism version 8.0.2. Differences between groups were evaluated using One-way ANOVA. Differences between samples were considered statistically significant at *p* < 0.05.

## 3. Results

### 3.1. CH-P treatment improves diabetes symptoms in mice

**Figure 1:**
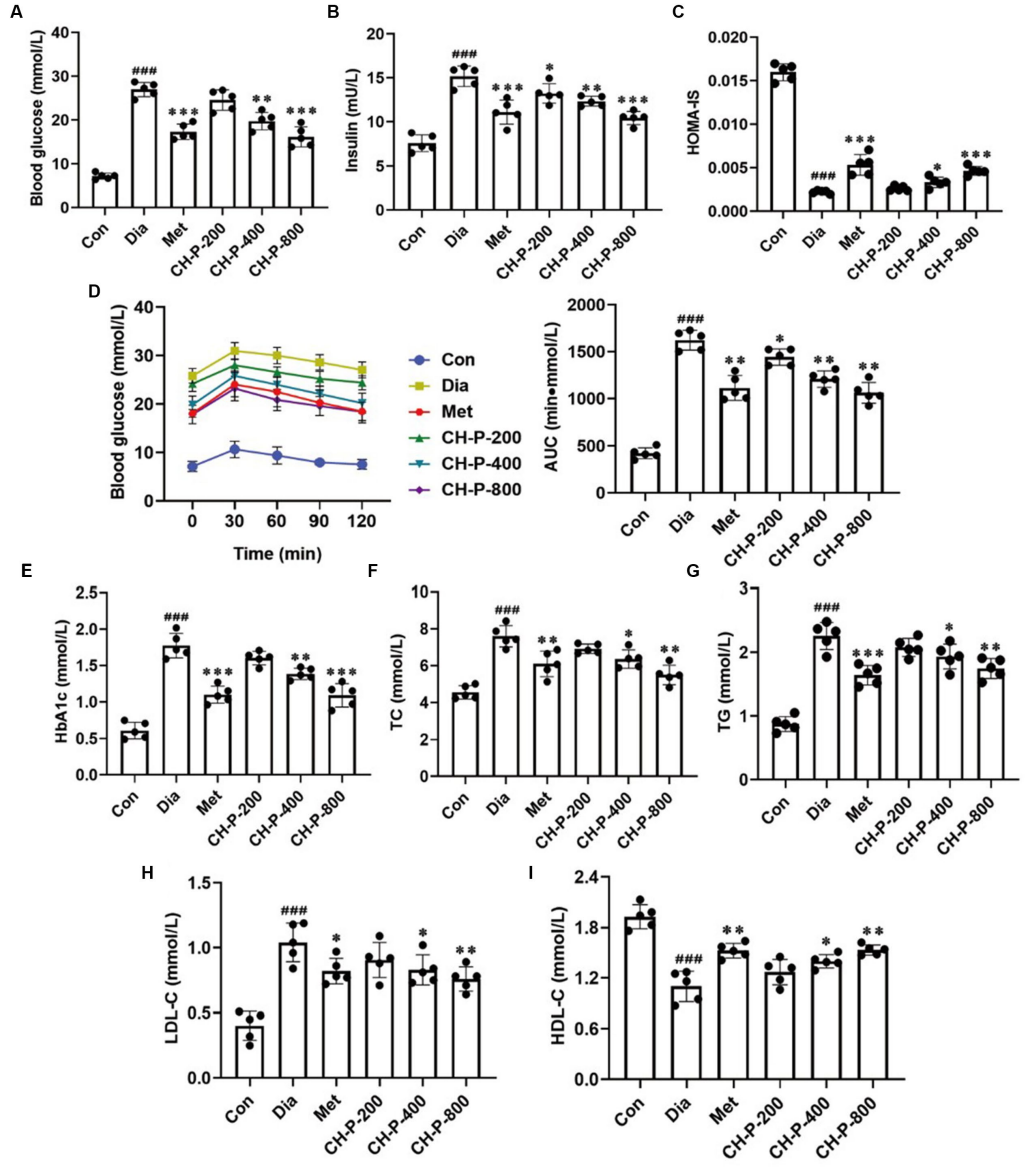
Analysis of CH-P treatment on hyperglycemia and dyslipidemia. **(A)** Blood glucose. **(B)** Insulin. **(C)** Homeostatic model assessment of insulin sensitivity (HOMA-IS). **(D)** Oral glucose tolerance test (OGTT) and area under the curve (AUC). **(E)** HbA1c. **(F)** Total cholesterol (TC). **(G)** Total triglycerides (TG). **(H)** Low-density lipoprotein-cholesterol (LDL-C). **(I)** High-density lipoprotein-cholesterol (HDL-C). ^###^*p* < 0.001, vs. Con; ^*^p < 0.05, vs. Dia; ^**^p < 0.01, vs. Dia; ^***^p < 0.001, vs. Dia.

Body weight, food intake, and water intake of diabetic mice significantly decreased, which was positively correlated with the concentration of polysaccharides purified from *C. cicadae* compared with the diabetic control group after 4 weeks of treatment (Supplementary Figures 1A–C). Moreover, *C. cicadae* polysaccharides significant reduction in blood glucose and insulin levels in a dose-dependent by decreased by 39.92 and 31.27% at a dosage of 800 mg/kg, respectively, and it also significantly improved the HOMA-IS relative to the diabetic control (9.63 ± 1.03 vs. 20.08 ± 1.66, *p* < 0.001; Figures 1A–C). The OGTT results showed that the changing trend in blood glucose was the same in all mice. However, the blood glucose tolerance level significantly improved in the CH-P treatment group compared with that in the diabetic control group. The area under the curve (AUC) values in the CH-P treatment group decreased in a dose-dependent manner, and exhibited up to a 35.56% decrease compared to the untreated diabetic mice (Figure 1D). Dyslipidemia is one of the main risk factors for diabetes and its associated complications. HbA1c, TG, TC, and LDL-C levels significantly increased, whereas HDL-C levels significantly decreased in diabetic mice compared with the control group. The CH-P treatment reversed these index values in a dose-dependent manner; there was a significant decrease of 39.76% HbA1c, 43.36% TG, 27.63% TC, and an increase of 28.10% HDL-C at a dose of 800 mg/kg compared with the diabetic control, respectively (Figures 1E–I). Overall, the therapeutic effect of *C. cicadae* polysaccharides at the dose of 800 mg/kg is better than that of metformin, but it was lower at the dose of 400 mg/kg than that of metformin.

### 3.2. Treatment with CH-P alleviated tissue damage in diabetes

**Figure 2:**
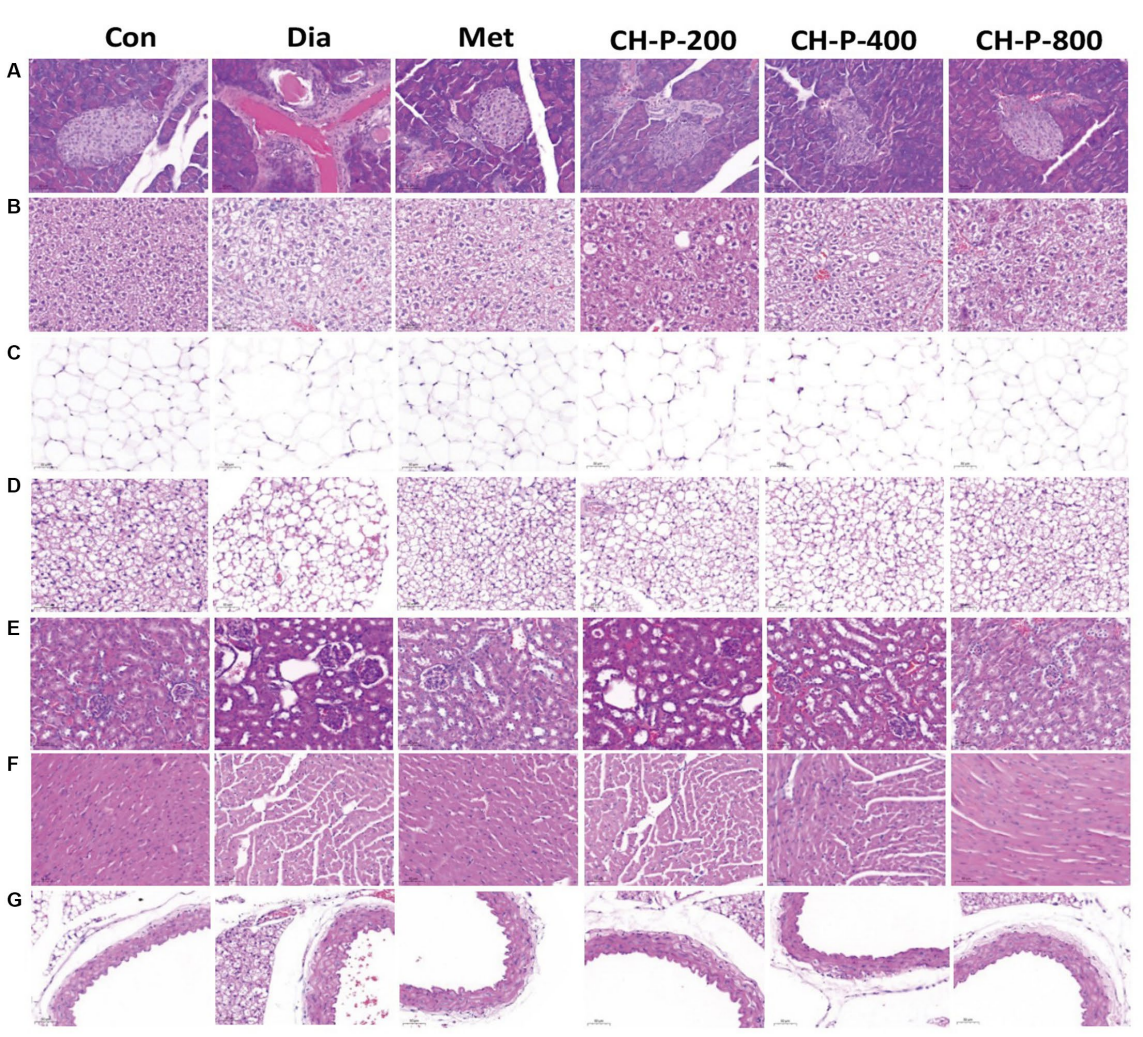
CH-P treatment recovers tissue damage caused by diabetes. **(A)** Pancreas. **(B)** Liver. **(C)** White fat. **(D)** Brown fat. **(E)** Kidney. **(F)** Heart. **(G)** Aorta. Bars represents 50 μm.

Long-term hyperglycemia in diabetes can lead to chronic injury and tissue dysfunction. The endocrine pancreas plays a central role in controlling blood glucose levels and regulating energy metabolism (19). The islets of diabetic mice showed contraction and distortion compared to the control group. The islet boundaries were blurred and the number of islet cells significantly decreased. Treatment with CH-P significantly increased the number of islets and islet cells. In addition, CH-P treatment made the boundaries of the islets clearer, and the shape and size of the islets were significantly restored (Figure 2A). Adipose tissue is classified as white, brown, or beige fat based on its anatomical location and metabolic functions. It is a major metabolic and endocrine organ that plays a key role in glucose and energy homeostasis (20). White adipose tissue is the main tissue for energy storage, and excessive white adipose tissue (WAT) is associated with hyperglycemia and insulin resistance. The functions of brown adipose tissue include energy consumption and heat production, which play important roles in the regulation of energy metabolism (21). This study showed that CH-P treatment significantly reversed the enlargement of white adipocyte tissue and adipose tissue increases associated with diabetes. In addition, CH-P treatment significantly increased the number of brown fat cells in diabetic mice, decreased the outline and boundaries of adipose cells, and decreased the degree of cell swelling (Figures 2B,C). The liver is an important metabolic organ that plays an important role in the maintenance of blood glucose homeostasis. Glucose metabolism disorders in the liver are involved in the development of T2DM (22). Diabetic mice show significant adipose vacuoles and cellular swelling. The group treated with CH-P showed significantly improved hepatocyte hypertrophy and lipid accumulation compared to those in diabetic mice (Figure 2D). Diabetic nephropathy is a serious complication of diabetes (23). CH-P treatment also improved renal lesions in diabetic mice, significantly reduced glomerular volume, and alleviated mesangial hyperplasia compared to diabetic mice (Figure 2E). Chronic hyperglycemia and diabetes lead to impaired cardiac function and an increased risk of arrhythmia (23). The cardiac tissue of diabetic mice exhibited myocardial hypertrophy with varying degrees of interstitial edema and histiocytosis and displayed inflammation and fibrosis of the endocardium or pericardium. CH-P treatment ameliorated myocardial hypertrophy and relieved tissue inflammation and fibrosis (Figure 2F). T2DM can induce ultrastructural damage to the tunica intima and tunica media of the aorta (24). The tunica media of the aortae was significantly thickened in diabetic mice and CH-P treatment significantly reversed the pathological damage (Figure 2G).

### 3.3. The effect of *Cordyceps cicadae* polysaccharides on antioxidant and anti-inflammation profiles

**Figure 3:**
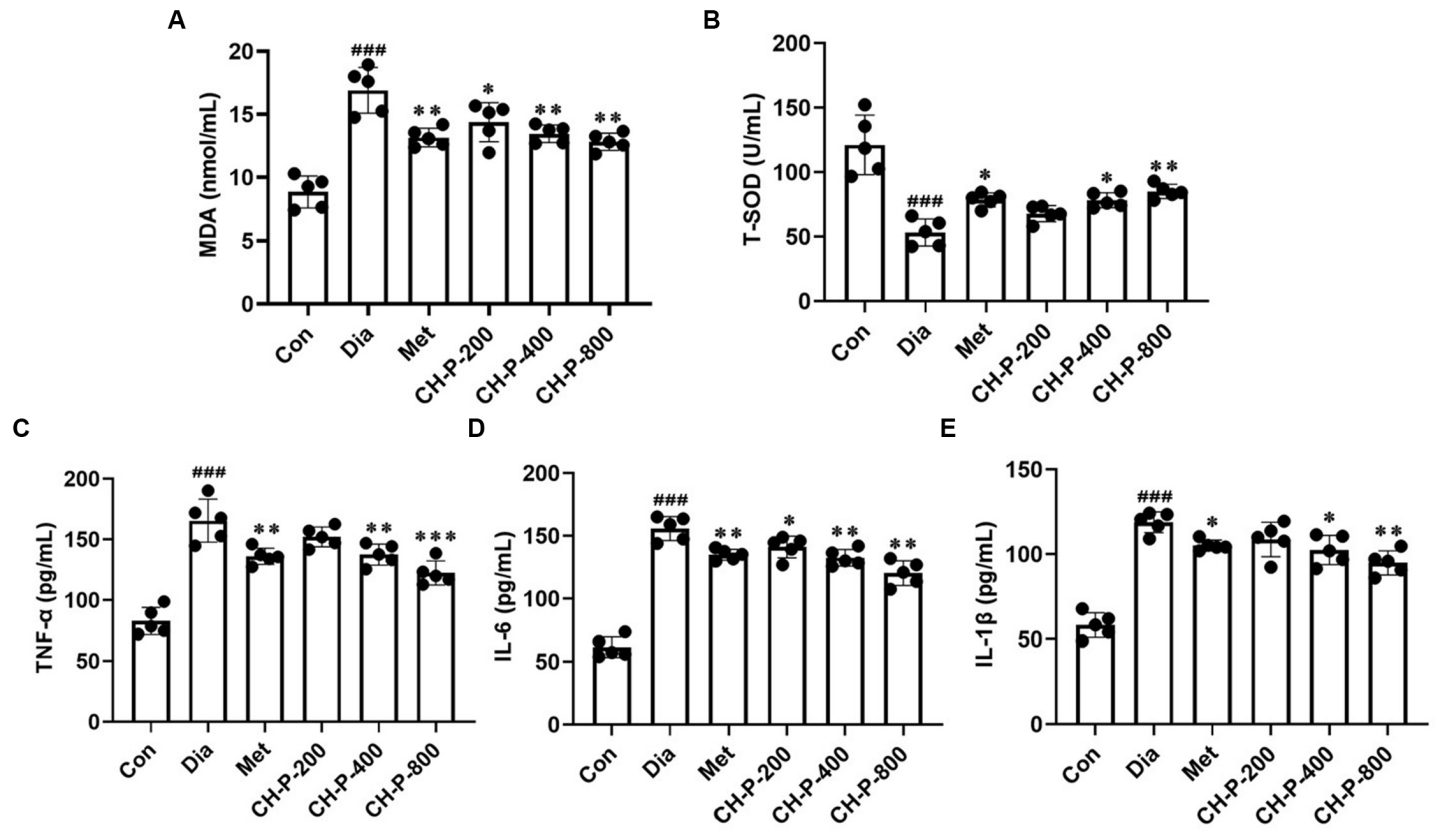
Analysis of the antioxidant and anti-inflammatory activities of CH-P. **(A)** MDA content. **(B)** T-SOD activity. **(C)** TNF-α. **(D)** IL-1β. **(E)** IL-6. ^###^*p* < 0.001, vs. Con; ^*^p < 0.05, vs. Dia; ^**^p < 0.01, vs. Dia; ^***^p < 0.001, vs. Dia.

Oxidative stress is the main factor in hyperglycemia-induced tissue injury and is responsible for events related to the early development of T2DM (25). The total antioxidant capacity of diabetic mice significantly decreases, which is consistent with the results of this study. However, CH-P treatment significantly decreased the MDA content and increased the T-SOD activity in the serum of diabetic mice in a dose-dependent manner; there was a 24.16% decrease in MDA and a 37.38% increase in T-SOD levels at a dose of 800 mg/kg relative to diabetic mice (Figures 3A,B).

Chronic inflammation is closely related to liver insulin resistance, and the progression of T2DM is often accompanied by chronic low-grade inflammation (26). The levels of circulating inflammatory markers such as proinflammatory cytokines significantly increased in diabetes and play an important role in the occurrence and development of T2DM (27). The effects of CH-P on serum pro-inflammatory cytokine levels were shown in Figure 3. CH-P treatment markedly reduced the levels of the pro-inflammatory cytokines TNF-α, IL-1β, and IL-6 in a dose-dependent manner compared to the high levels of pro-inflammatory cytokines in the diabetic control group. TNF-α, IL-1β, and IL-6 decreased by 26.09, 20.13, and 22.85%, respectively at a dose of 800 mg/kg CH-P compared with the diabetic control (Figures 3C–E).

### 3.4. The gut microbiota composition changes following treatment with *Cordyceps cicadae* polysaccharide

**Figure 4:**
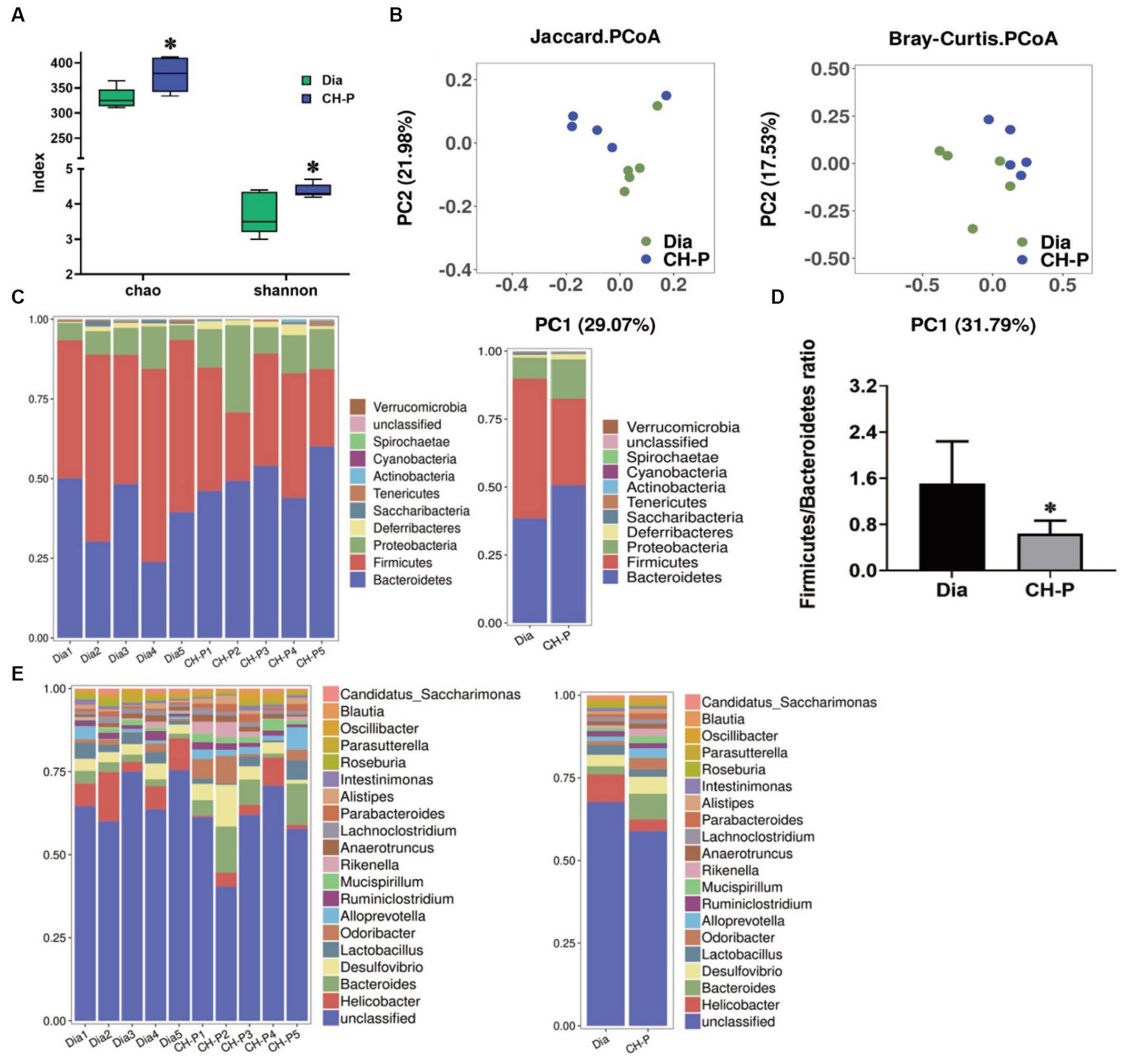
Gut microbiota diversity and composition analysis. **(A)** The alpha diversity indexes of gut microbiota for richness (Chao index) and evenness (Shannon index) between the two groups. **(B)** Principal component analysis of the gut microbiota between the diabetic and CH-P treatment group based on Jaccard and Bray-Curtis. **(C)** Relative abundance of gut microbiota at the phylum level. **(D)** The ratio of Firmicutes/Bacteroidetes. **(E)** Relative abundance of fecal microbiota at the genus level. ^*^p < 0.05 indicated significant differences.

Perturbations in the gut microbiota can influence the development of some metabolic disorders, (including obesity and diabetes) through different metabolic and immune pathways, including anti-inflammatory status, abnormal glucose metabolism, and insulin resistance (28, 29). 16S rDNA sequencing was used to detect the fecal microbiota composition and changes in mice to explore whether CH-P exerts a hypoglycemic effect by modulating intestinal microorganisms. The alpha diversity significantly increased in the CH-P treatment group compared to the diabetic group according to the Chao (376.6 ± 34.52 vs. 329.2 ± 20.83, *p* < 0.05) and Shannon indices (4.38 ± 0.19 vs. 3.72 ± 0.61, *p* < 0.05; Figure 4A). This suggests that the CH-P treatment boosts species richness and species evenness of gut microbiota in diabetic mice. Moreover, PCoA based on Jaccard and Bray-Curtis showed that the microbial communities of the two groups were clearly separated and clustered (Figure 4B). This indicated that CH-P treatment significantly influenced the gut microbiota composition. To investigate the changes in fecal microflora in response to CH-P treatment, the gut microbiota was analyzed at different taxonomic levels. The relative abundance of phyla levels of *Firmicutes*, *Bacteroidetes*, *Proteobacteria,* and *Desulfobacterota* was primarily dominated in both groups, and more than 90% of the total bacteria were comprised of *Firmicutes* and *Bacteroidetes* (Figure 4C). The *Firmicutes*/*Bacteroidetes* ratio was significantly reduced in the CH-P treatment group compared to the diabetic group (Figure 4D). An increased ratio of *Firmicutes*/*Bacteroidetes* and changes in several bacterial species can promote the development of obesity in dietary and genetic models of obesity in mice (30). These results suggest that CH-P has a beneficial effect on the health of intestinal microbiota. The top 20 gut genera microbiota were analyzed to determine the relative abundance differences between the two groups. The CH-P treatment significantly increased the abundance of *Bacteroides*, *Odoribacter*, *Alloprevotella*, *Parabacteroides*, and *Mucispirillum*, and significantly decreased the abundance of *Helicobacter* and *Lactobacillus* compared to the diabetic group (Figure 4E). The relative abundance of class, order, family, and species levels were showed in Supplementary Figure 2.

**Figure 5:**
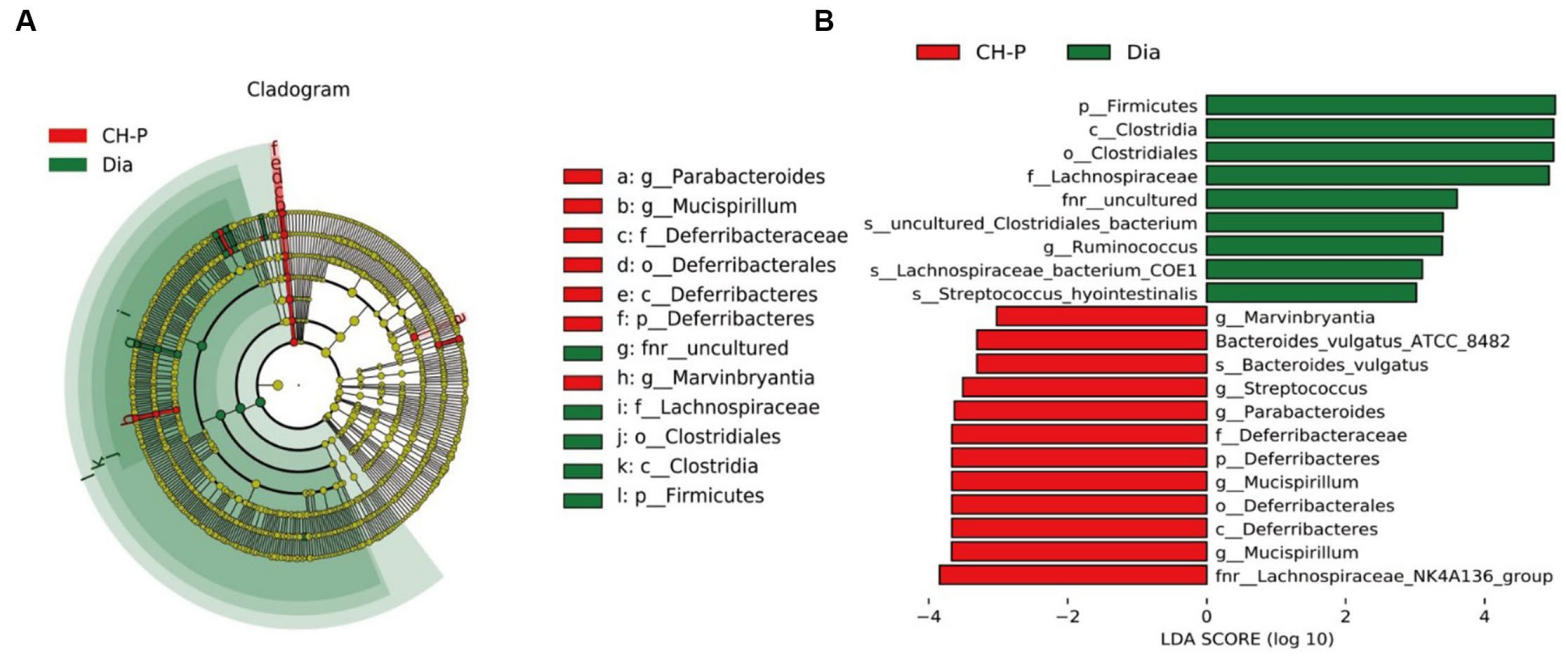
Linear discriminant analysis Effect Size (LEfSe). **(A)** Cladogram indicating the phylogenetic distribution of microbiota correlated with the diabetic or CH-P treatment groups. **(B)** The difference in abundance between the diabetic and CH-P treatment groups.

Linear discriminant analysis effect size analysis was performed to generate a cladogram to identify different biomarkers in the gut microbiota composition of the two groups. There were distinguishing components at different taxon levels, including 12 species enriched in the CH-P group and nine species enriched in diabetic mice (Figure 5). The decreased abundance in *Firmicutes* was primarily related to a decrease in *Clostridiales*, *Lachnospiraceae*, *Ruminococcus*, and *Streptococcus hyointestinalis* in CH-P-treated mice. Meanwhile, the increased abundance of *Bacteroidetes* was mainly attributed to an increase in *Parabacteroides* and *Bacteroides vulgatus*. Moreover, the CH-P treatment showed desirable effects in restoring other key bacterial species in diabetic mice including *Mucispirillum* (which significantly increased from the phylum to the genus level) and *Marvinbryantia* (Figure 5).

### 3.5. The effect of CH-P treatment on the metabolism of diabetic mice

**Figure 6:**
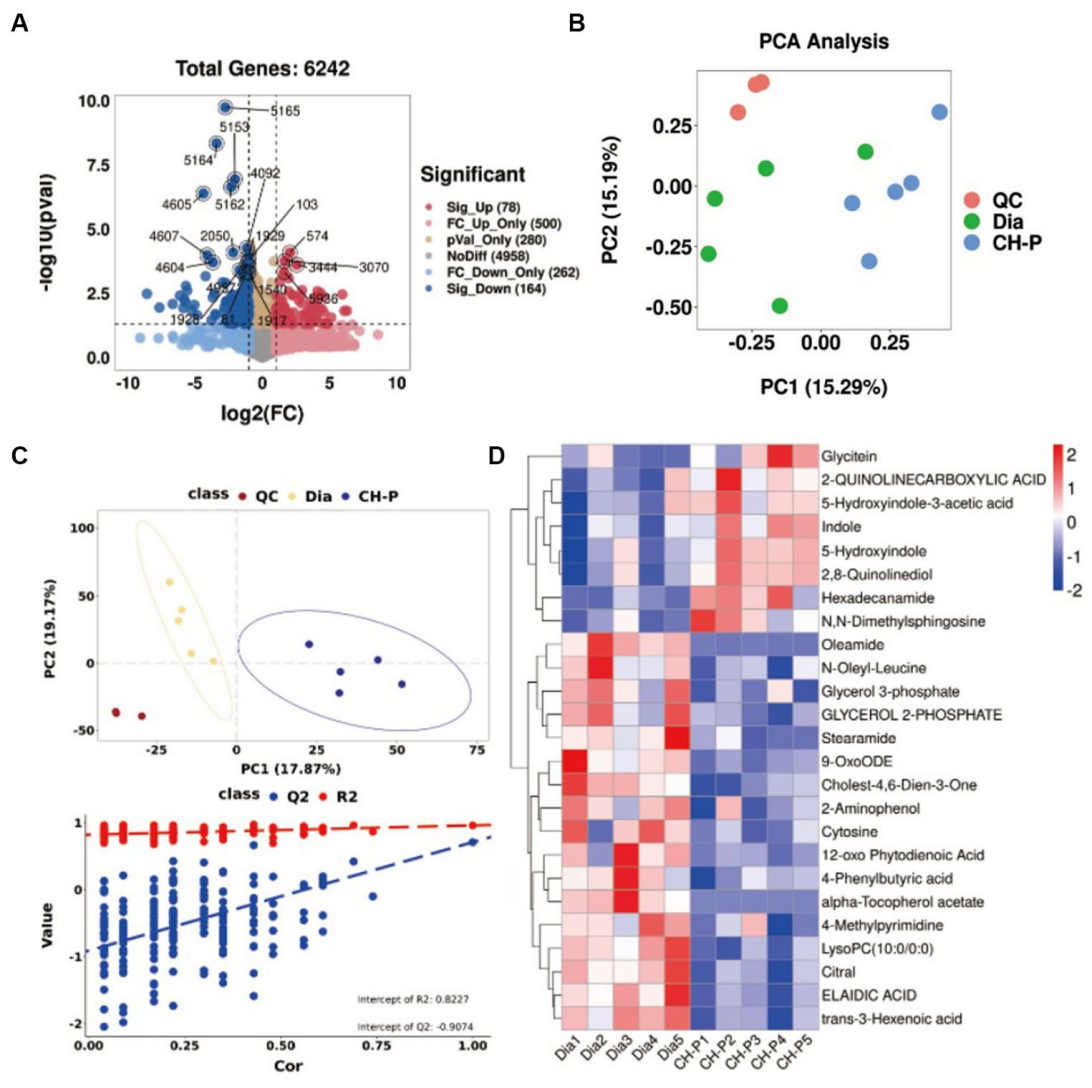
Untargeted metabolomics analysis of the metabolites in the diabetic and CH-P treatment groups using the POS model. **(A)** Principal component analysis score plot showing comparisons of the metabolomic profiles in both groups. **(B)** Volcano plot showing the differentially accumulated and significantly changed metabolites. **(C)** The PLS-DA score plot comparing the metabolome profiles in both groups and statistical validation of the PLS-DA model by permutation testing. **(D)** Hierarchical clustering heatmap of metabolites showing metabolites that significantly differ in abundance between both groups.

The gut microbiota exerts important and diverse effects on host physiology by generating metabolites with health benefits (31). Therefore, an untargeted gas chromatography–mass spectrometry (GC–MS)-based metabolomic analysis was performed to study the effect of CH-P on the metabolic profiles of mouse fecal samples. A total of 6,242 (POS) and 4,760 (NEG) metabolites were detected (Figure 6A; Supplementary Figure 3A). The quality control (QC) samples clustered in the PCA plots demonstrate the stability and repeatability of the acquisition method (Figure 6B; Supplementary Figure 3B). The PCA plots showed that the metabolomics in the diabetic and CH-P groups showed a clear separation trend (Figure 6B; Supplementary Figure 3B). Partial least-squares discriminant analysis (PLS-DA) showed that the CH-P treatment significantly altered the metabolic profiles of diabetic mice. The permutation test of the PLS-DA model showed good stability and no overfitting (Figure 6C; Supplementary Figure 3C). The upregulated and downregulated metabolomics revealed that there were 38 significantly changed metabolites (25 POS and 13 NEG) between the diabetic and CH-P groups visualized by the heat maps (Figure 6D; Supplementary Figure 3D).

**Figure 7:**
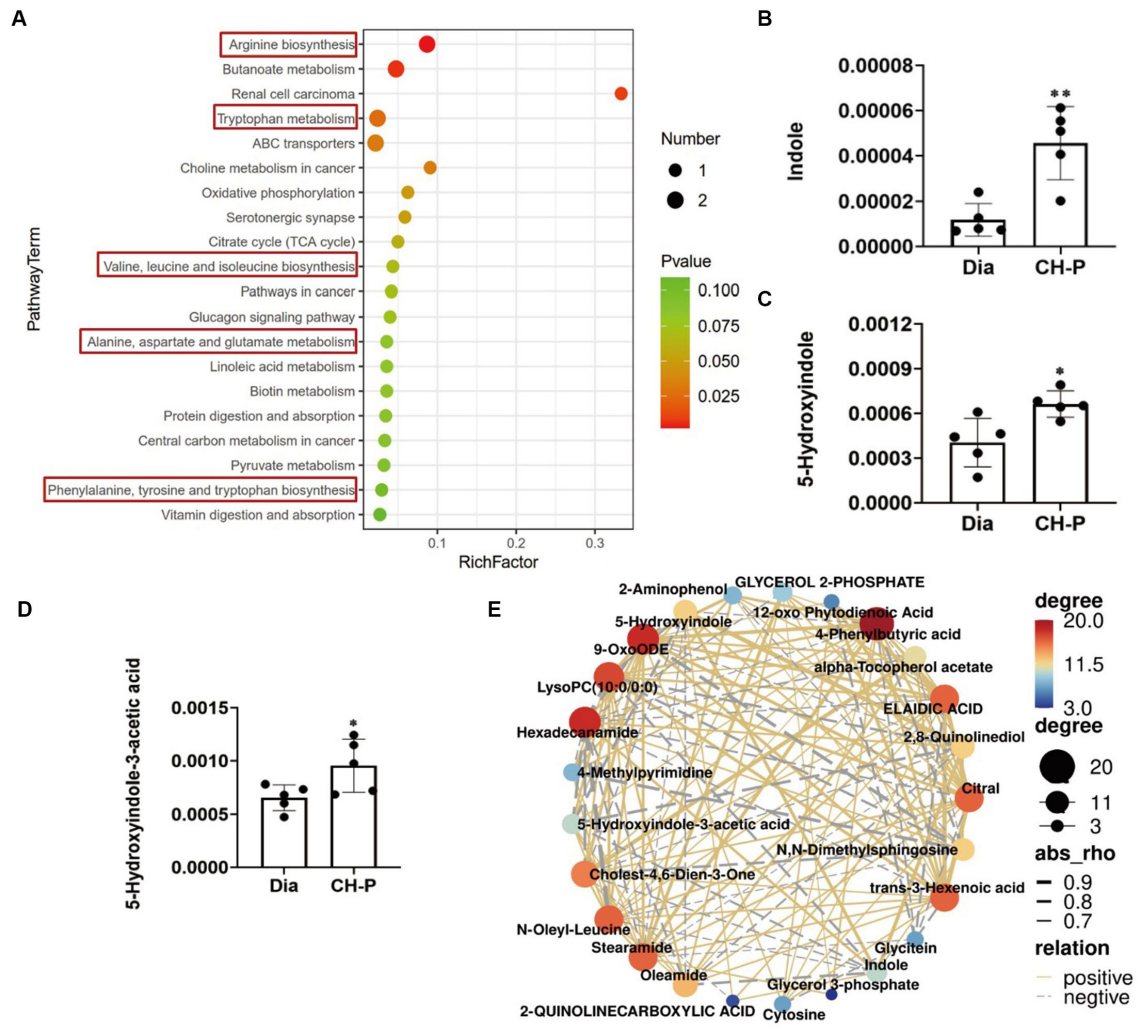
Metabolic pathways involving the differential metabolites and their correlation. **(A)** Pathway enrichment analysis of the differential metabolites. **(B)** Comparison of the relative abundance of indole in diabetic and CH-P treatment groups. **(C)** Comparison of the relative abundance of 5-hydroxyindole in diabetic and CH-P treatment groups. **(D)** Comparison of the relative abundance of 5-hydroxyindole-3-acetic acid in diabetic and CH-P treatment groups. **(E)** Correlation analysis of the differential metabolites using the POS model. ^*^p < 0.05, vs. Dia; ^**^p < 0.01, vs. Dia.

The metabolic pathways involving the altered metabolites were determined using KEGG pathway enrichment analysis, and the top 20 metabolic pathways are shown in Figure 7A and Supplementary Figure 4A. Interestingly, pathway enrichment analysis indicated that major alterations in metabolic pathways were related to amino acid biosynthesis and metabolic pathways, including arginine biosynthesis; tryptophan metabolism; valine, leucine, and isoleucine biosynthesis; and phenylalanine, tyrosine, and tryptophan biosynthesis. Therefore, we speculated that CH-P could improve diabetes by regulating the production of metabolites related to amino acid biosynthesis and metabolism, particularly tryptophan biosynthesis and the metabolic pathways of gut microorganisms (Figure 7A). Indole and its derivatives (such as 5-hydroxyindole) are core intermediates of tryptophan metabolism (32). CH-P treatment significantly improved the relative abundance of indole, 5-hydroxyindole, and 5-hydroxyindole-3 acetic acid compared to that of diabetic mice (Figures 7B–D). Differential metabolite correlation analysis was performed to analyze the correlations between individual metabolites. The metabolites correlated well with each other and the relationships between various metabolites are shown in Figure 7E and Supplementary Figure 4B. The abundance of indole and its derivatives were significantly positively correlated. This discovery may provide a new theory for the therapeutic mechanisms of polysaccharides from *C. cicadae*.

### 3.6. Potential relationship analysis between gut microbiota and metabolites in diabetic mice

**Figure 8:**
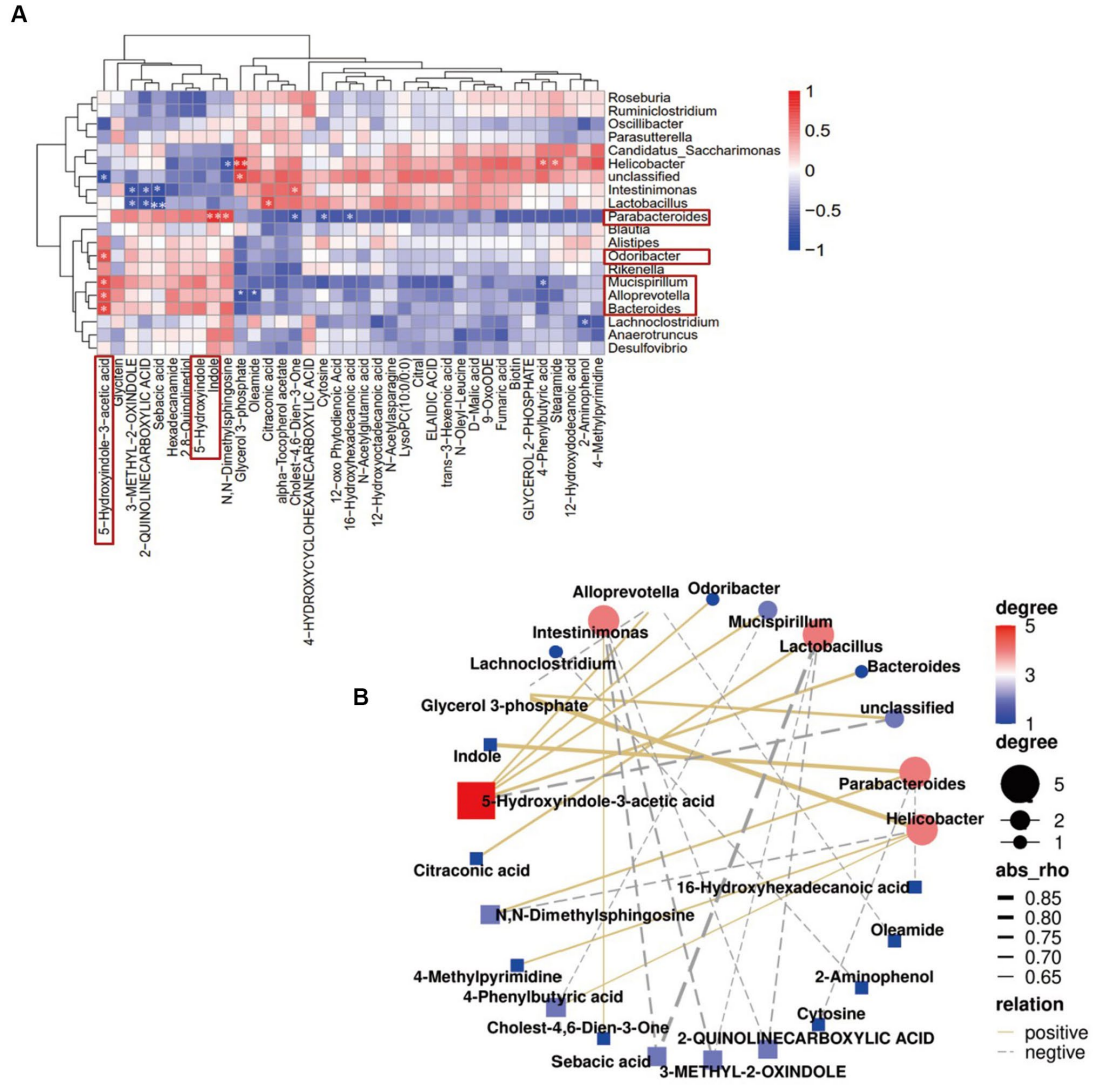
Correlation analysis of dominant gut microbiota genera with the differential metabolites. **(A)** Hierarchical clustering heatmap showing the correlation between dominant gut microbiota genera and the differential metabolites. The color intensity shows the degree of correlation (red and blue represents a positive and negative correlation, respectively). ^*^p < 0.05 and ^**^p < 0.01 indicated significant correlations. **(B)** Network map illustrating interactions among the dominant gut microbiota genera and the differential metabolites.

Spearman’s correlation analysis was used to explore the potential relationship between gut microbiota and fecal metabolites. *Bacteroides*, *Odoribacter*, *Alloprevotella*, and *Mucispirillum* were positively correlated with 5-hydroxyindole-3-acetic acid, while *Alloprevotella* was negatively correlated with glycerol 3-phosphate and oleamide. *Parabacteroides* was positively correlated with indole and N, N-dimethylsphingosine, and negatively correlated with cholest-4,6-dien-3-one, cytosine, and 16-hydroxyhexadecanoic acid. Interestingly, indole is an important component involved in tryptophan metabolism and phenylalanine, tyrosine, and tryptophan biosynthesis, while 5-hydroxyindole-3-acetic acid is involved in tryptophan metabolism (Figure 8). These results were consistent with those of the KEGG pathway enrichment analysis. Together, the results showed that CH-P treatment can improve diabetes by affecting the abundance of gut microorganisms such as *Bacteroides*, *Odoribacter*, *Alloprevotella*, *Paraberoides*, and *Mucispirillum*. The gut microbiota can produce metabolites such as indole and 5-hydroxyindole-3-acetic acid to participate in tryptophan metabolism and phenylalanine, tyrosine, and tryptophan biosynthesis, and play a role in treating diabetes.

### 3.7. Effects of indole intervention and FMT on alleviating diabetes

**Figure 9:**
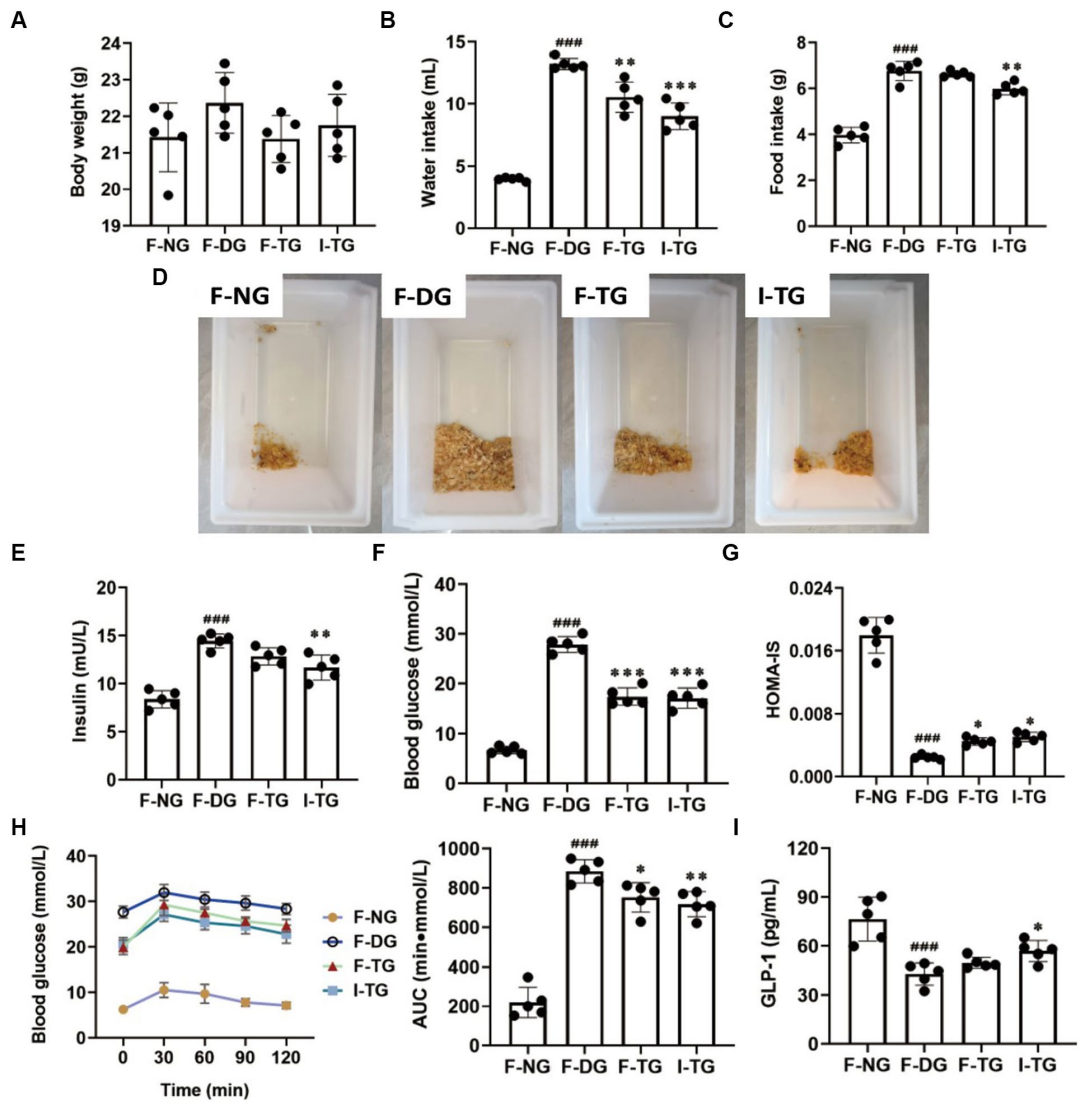
Effects analysis of indole intervention and FMT on alleviating diabetes. **(A)** Body weight. **(B)** Water intake. **(C)** Food intake. **(D)** Urination analysis. **(E)** Insulin. **(F)** Blood glucose. **(G)** HOMA-IS. **(H)** OGTT and AUC. **(I)** GLP-1. ^###^*p* < 0.001, vs. F-NG; ^*^p < 0.05, vs. F-DG; ^**^p < 0.01, vs. F-DG; ^***^p < 0.001, vs. F-DG.

Fecal microbiota transplantation experiments were performed by gavaging fecal suspensions from diabetic or CH-P-treated mice into reconstructed diabetic mice to further evaluate whether the gut microbiota and their metabolites exert hypoglycemic effects. Indole intervention was also performed to verify the effects of the differential metabolites. There was no significant difference in body weight; however, the water intake, urinary output, or blood glucose AUC of F-TG and I-TG mice significantly decreased compared to those of F-DG mice (Figures 9A,B,D,F,H). Moreover, they significantly improved insulin sensitivity compared to F-DG mice. However, indole treatment significantly reduced food intake and insulin concentration (Figures 9C,E). KEGG pathway analysis showed that the gut microorganisms that play a hypoglycemic role in the CH-P treatment group may produce metabolites through amino acid biosynthesis and metabolic pathways, especially tryptophan biosynthesis and metabolism (Figure 7A). Tryptophan is an essential aromatic amino acid that can be decomposed into indole and its derivatives, which have various beneficial effects on diabetes via gut microbiota (33). Interestingly, CH-P treatment significantly and positively correlated with the indole, 5-hydroxyindole, and 5-hydroxyindole-3-acetic acid metabolites. Indole modulates glucagon-like peptide-1 (GLP-1) to regulate postprandial glucose levels by stimulating insulin secretion and inhibiting glucagon secretion (34, 35). This study found that GLP-1 secretion significantly increased in I-TG mice compared to that in the F-DG group (Figure 9I). GLP-1 expression did not significantly increase in the F-TG mice; however, it showed an upward trend. This indicates that the polysaccharides from *C. cicadae* may alleviate hyperglycemia by regulating the production of metabolites other than indole and its derivatives by the gut microbiota.

## 4. Discussion

Type 2 diabetes mellitus is a metabolic disease characterized by hyperglycemia resulting from insulin resistance or deficiency (8). Controlling blood glucose levels is an effective method to treat and prevent diabetes and various complications associated with diabetes. Naturally, active polysaccharides were proposed as alternative therapeutic agents for T2DM. Polysaccharides from *S. thunbergii* have many pharmacological effects, including antioxidant and hypoglycemic properties (36). *Ganoderma atrum* polysaccharides display hypoglycemic activity by reducing fasting blood glucose levels, increasing endothelium-dependent aortic relaxation, and improving PI3K, AKT, eNOS, and NO levels in diabetic rats (37). This study found that CH-P significantly reduced blood glucose levels, and increased insulin sensitivity in diabetic mice (Figures 1A–C). In addition, CH-P improved oral glucose tolerance in diabetic mice and alleviated hyperglycemia-induced tissue damage (Figures 1D, 2).

Dyslipidemia is a common feature of diabetes and manifests as elevated plasma levels of TC, TG, and LDL-C and/or decreased HDL-C levels (38). Current evidence supports the beneficial effects of polysaccharides on blood lipid levels in T2DM patients. Administration of *Acanthopanax senticosus* polysaccharides improves hyperglycemia by decreasing TC, TG, and LDL levels in alloxan-induced diabetic mice (39). Polysaccharide-simulated hydrolysates obtained from the dried fruiting body of *Auricularia auricular* significantly decrease serum TG and LDL-C levels in diabetic rats (40). Similarly, this study showed that CH-P significantly reduced TC, TG, and LDL-C levels and increased HDL-C levels in diabetic mice (Figures 1E–I). This indicated that CH-P could be used as an alternative therapeutic agent to alleviate cardiovascular complications.

Oxidative stress is implicated in the development of impaired glucose tolerance, insulin resistance, and islet cell dysfunction in T2DM (41). Many studies demonstrate that fungal polysaccharides have remarkable antioxidant effects. *Tricholoma mongolicum Imai* polysaccharides display strong antioxidant activity by scavenging DPPH and hydroxyl radicals (•OH) radicals *in vitro*. *Auricularia auricular* polysaccharides significantly protect against exhaustive swimming exercise-induced oxidative stress by regulating the levels of SOD, MDA, glutathione peroxidase, and catalase in mice (42). Moreover, polysaccharide from seeds of *Plantago asiatica* L. improved the antioxidant capacity by increasing SCFAs-producing strain (43). Evidence indicates gut microbiota enhanced the antioxidant capacity by increasing the high-antioxidant molecules, reactive sulfur species, such as hydrogen sulfide and cysteine persulfide (44). This study found that CH-P significantly reduced serum MDA content and increased SOD activity in diabetic mice (Figures 3A,B), indicated that CH-P may exert antioxidant effects f by regulating gut microbial composition.

Chronic inflammation significantly contributes to the development of T2DM by promoting insulin resistance and β-cell failure (45, 46). Adolescents with T2DM have significantly higher concentrations of hsCRP, TNF-α, and IL-1β inflammatory markers (47). Decreased insulin sensitivity in T2DM is related to inflammatory mediators such as TNF-α, IL-1β, IL-6, IL-8, and MCP-1 (48). Inflammatory markers are considered therapeutic parameters for the development of T2DM and its complications, and targeted modulation of the inflammatory system is proposed as a therapeutic strategy for T2DM (49). Fungal polysaccharides are currently regarded as a powerful active ingredient to regulate inflammation (50, 51). This study showed that CH-P significantly reduced the levels of TNF-α, IL-1β, and IL-6 in serum (Figures 3C–E). This shows that CH-P can alleviate diabetes by reducing inflammatory factors.

An aberrant gut microbiota composition is highly correlated with obesity and insulin resistance, which play important roles in the onset and development of T2DM (52). *Enterobacter cloacae* is the causative gut bacterium that induces obesity and insulin resistance in animals (53). The percentage of butyrate-producing bacteria (such as *Roseburia* and *Faecalibacterium prauznitzii*) is significantly reduced in the gut microbiota of patients with T2DM (54). However, there are increased levels of *Lactobacillus* and some opportunistic pathogens such as *Desulfovibrio* sp. and *Clostridium* (55, 56). Therefore, regulating the composition of intestinal microorganisms may be a new strategy to improve T2DM. *Bacteroidetes*, *Firmicutes*, and *Actinobacteria* are the three major phyla responsible for degrading complex nondigestible polysaccharides (57). *Firmicutes*, *Bacteroidetes*, *Proteobacteria*, and *Desulfobacterota* phyla were the dominant bacterial communities in the CH-P treatment group and the diabetic model group (Figure 4C). CH-P treatment significantly reduced the *Firmicutes*/*Bacteroidetes* ratio (Figure 4D). This is consistent with previous studies (58, 59) and indicated that CH-P regulated the abundance and composition of intestinal microorganisms to improve diabetes. The decreased abundance in *Firmicutes* was primarily related to a decrease in *Clostridiales*, *Lachnospiraceae*, *Ruminococcus*, and *Streptococcus hyointestinalis* in CH-P-treated mice. Meanwhile, the increased abundance of *Bacteroidetes* was mainly attributed to an increase in *Parabacteroides* and *Bacteroides vulgatus*. Moreover, the CH-P treatment showed desirable effects in restoring other key bacterial species in diabetic mice including *Mucispirillum* and *Marvinbryantia* (Figure 5). In addition, CH-P treatment significantly increased the abundance of *Bacteroides*, *Odoribacter*, *Alloprevotella*, and *Parabacteroides* genera and significantly decreased the abundance of *Helicobacter* and *Lactobacillus* (Figure 4E). *Bacteroides* is a common SCFA-producing gut microbiota that benefits humans by maintaining an anaerobic intestinal luminal environment (60). An increased abundance of SCFA-producing- and anti-inflammatory bacterium *Alloprevotella* improves the symptoms of type 2 diabetic rats (61). *Parabacteroides distasonis* alleviates obesity and metabolic dysfunction by producing succinate and secondary bile acids (62). Intermittent fasting increases the levels of butyrate-producing *Odoribacter* to alleviate diabetes-induced cognitive impairment (63). These results indicate that CH-P can improve diabetes by changing the abundance and composition of intestinal microorganisms. However, the function and mechanism of these bacteria require further study.

Disease states can be ameliorated or prevented by reshaping the gut microbiota. In other words, inoculation of the host with intestinal microbiota can lead to durable metabolic changes with therapeutic utility (64). Many interventional studies show that the intake of intestinal probiotics improves intestinal microecological disorders and relieves the symptoms of patients with diabetes. Administration of polysaccharides from *Enteromorpha prolifera* ameliorates HFD-induced gut dysbiosis by modulating the composition of gut microbiota, such as *Bacteroides*, *Parabacteroides*, *Alloprevotella*, *Ruminococcus*, and gut barrier-protective *Akkermansia muciniphila*, which resulted in an enrichment of the related metabolites of SCFA, and a reduction in circulating lipopolysaccharide (LPS) levels (65). *Parabacteroides distasonis* alleviates obesity and metabolic dysfunction by producing succinate and secondary bile acids. Carnosic acid exerts anti-inflammatory effects against colitis by altering the gut microbiota and correlated metabolites (66). Specific gut microbes can transform some amino acids into various bioactive metabolites (29). This study showed that the alterations in the metabolic pathways were mostly related to amino acid biosynthesis and metabolic pathways, particularly those involving tryptophan. Tryptophan metabolism plays a role in the gastrointestinal tract through three major pathways: (1) Tryptophan is directly decomposed by microorganisms into small molecule ligands that can bind to aryl hydrocarbon receptors or other receptors; (2) Kynurenine pathway; and (3) Serotonin pathway (33). Tryptophan can be metabolized to indole by several bacterial species, such as *Bacteroides thetaiotaomicron*, *B. ovatus*, *Clostridium limosum*, and *C. bifermentans* (32). Indole, as an interspecies signaling molecule, plays important modulate roles in bacterial pathogenesis and eukaryotic immunity, such as antibiotic resistance, oxidative stress, intestinal inflammation, and hormone secretion (67). Moreover, indole and its derivatives such as indole-3-propionic acid (IPA) have various beneficial effects on diabetes. Indole modulates the secretion of glucagon-like peptide-1 (GLP-1) from intestinal enteroendocrine L-cells (34). The main role of GLP-1 is to stimulate insulin secretion and inhibit glucagon secretion, thereby improving postprandial glucose fluctuations (35). This study showed that CH-P treatment significantly increased the metabolites of indole, 5-hydroxyindole and 5-hydroxyindole-3-acetic acid (Figures 6D, 7). Spearman’s correlation analysis showed that *Bacteroides*, *Odoribacter*, *Alloprevotella*, and *Mucispirillum* were positively correlated with 5-hydroxyindole-3-acetic acid, while *Parabacteroides* was positively correlated with indole and N, N-dimethylsphingosine. Indole is an important component involved in tryptophan metabolism and phenylalanine, tyrosine, and tryptophan biosynthesis, while 5-hydroxyindole-3-acetic acid is involved in tryptophan metabolism (Figure 8). Together, the results showed that CH-P treatment can improve diabetes by affecting the abundance of gut microorganisms such as *Bacteroides*, *Odoribacter*, *Alloprevotella*, *Paraberoides*, and *Mucispirillum*. The gut microbiota can produce metabolites such as indole and 5-hydroxyindole-3-acetic acid, and modulated GLP-1 secretion to improve diabetes. Therefore, this study verified the hypoglycemic effect of indole. Indole significantly improved the hyperglycemic symptoms and insulin sensitivity of mice, including decreased water intake, food intake, urinary output, insulin level, blood glucose, and glucose tolerance compared to F-DG (Figures 9B–H). More importantly, indole significantly increased GLP-1 secretion (Figure 9I). This confirmed that gut microbiota produces metabolites such as indole and its derivatives and modulates the secretion of GLP-1 to alleviate the effects of diabetes.

Fecal microbiota transplantation improves insulin resistance and pancreatic islet β-cell function by reconstructing the gut microbiota (68). Moreover, it alters the susceptibility of obese rats to T2DM (69). This study used FMT to reconstruct the gut microbiota in high-fat diet/STZ-induced diabetic mice. FMT successfully reduced blood glucose levels and improved glucose tolerance in diabetic mice (Figures 9F,H). The insulin sensitivity in the F-TG group increased after T2DM mice reconstructed their microbiota by administering the feces of CH-P-treated mice (Figure 9G). Similarly, a clinical trial showed that obese patients receiving FMT from lean, healthy individuals showed a positive change in insulin sensitivity (70). However, FMT did not significantly improve GLP-1 levels (Figure 9I). This indicates that *C. cicadae* polysaccharides alleviated hyperglycemia by regulating the production of metabolites other than indole and its derivatives by gut microbiota. However, CH-P treatment increased the levels of SCFA-producing bacteria (*Bacteroides*, *Odoribacter*, *Alloprevotella*, and *Parabacteroides*).Further investigation is required to determine whether CH-P plays a therapeutic role in diabetes through the production of SCFA. In addition, this work did not analyze the structural characteristics of CH-P, which requires further in-depth explore in the future.

In addition to a variety of pharmacological activities, *C. cicadae* also have the advantages of low toxicity, low price and easy artificial cultivation. At present, a wide variety of functional products and health care products are produced from *C. cicadae*, covering health food, cosmetics, biological agriculture, and pharmaceutical fields (71). However, researches on *C. cicadae* are limited to preliminary pharmacochemical and pharmacological studies. Therefore, the further exploration of *C. cicadae* polysaccharide in this study is expected to expand its application scope and better play its health care effect.

## Data availability statement

The original contributions presented in the study are publicly available. This data can be found at: https://www.ncbi.nlm.nih.gov/bioproject/PRJNA954535.

## Ethics statement

All animal experimental protocols were reviewed and approved by the Experimental Animal Ethics Committee of Xuzhou Medical University (approval number: L20210226457).

## Author contributions

AC contributed to conception and design of the study. YW, ZN, JL, YS, YY, and WL performed the experiments. SZ and TF performed the data analysis. ZN wrote the manuscript. All authors contributed to the article and approved the submitted version.

## Funding

This work was supported by Jiangsu Province’s industry university research cooperation project (grant numbers BY2022773 and BY2022777); Xuzhou Science and Technology Program (grant numbers KC21273, KC21119, and KC22477); and Innovation Training Program for College Students of Xuzhou University of Technology (grant number xcx2023218).

## Conflict of interest

The authors declare that the research was conducted in the absence of any commercial or financial relationships that could be construed as a potential conflict of interest.

## Publisher’s note

All claims expressed in this article are solely those of the authors and do not necessarily represent those of their affiliated organizations, or those of the publisher, the editors and the reviewers. Any product that may be evaluated in this article, or claim that may be made by its manufacturer, is not guaranteed or endorsed by the publisher.
