# Supplementary material for: Cordyceps cicadae polysaccharides alleviate hyperglycemia by regulating gut microbiota and its mmetabolites in high-fat diet/streptozocin-induced diabetic mice
Source: Front Nutr. 2023 Aug 3;10:1203430. doi: 10.3389/fnut.2023.1203430 (PMC10434777; doi:10.3389/fnut.2023.1203430)
Supplement: Supplementary file 1 [file Data_Sheet_1.docx]

Supplementary Material

***Cordyceps cicadae* polysaccharides alleviate hyperglycemia by regulating gut microbiota and its metabolites in mice**

**Yanan Wang^1^, Zaizhong Ni^1^, Jinting Li^1^, Ying Shao^1^, Yidan Yong^1^, Wendi Lv^1^, Simeng Zhang^1^, Tingwei Fu^1^, Anhui Chen^1*^**

*** Correspondence:** Anhui Chen: chenah201@163.com

# Supplementary Data

Supplementary Material should be uploaded separately on submission. Please include any supplementary data, figures and/or tables.

Supplementary material is not typeset so please ensure that all information is clearly presented, the appropriate caption is included in the file and not in the manuscript, and that the style conforms to the rest of the article.

# Supplementary Figures and Tables

For more information on Supplementary Material and for details on the different file types accepted, please see [here](https://www.frontiersin.org/guidelines/author-guidelines#supplementary-material).

## Supplementary Figures


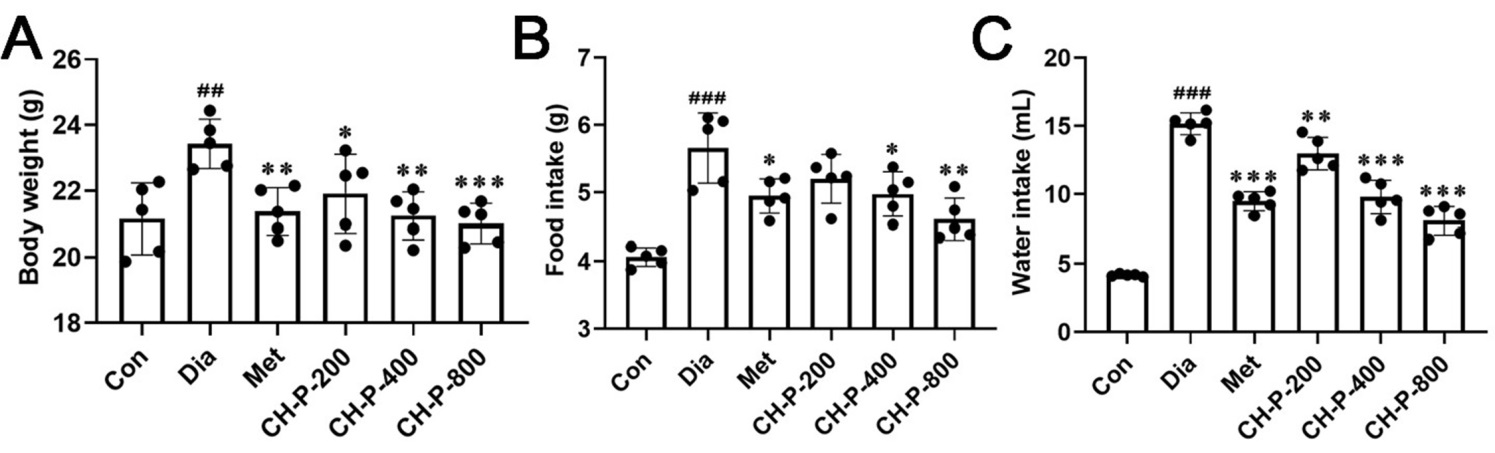


**Supplementary Figure 1.** Analysis of CH-P treatment on (A) body weight, (B) food intake, and (C) water intake. ###, p<0.001, vs. Con; ##, p<0.01, vs. Con; *, p<0.05, vs. Dia; **, p<0.01, vs. Dia; ***, p<0.001, vs. Dia.


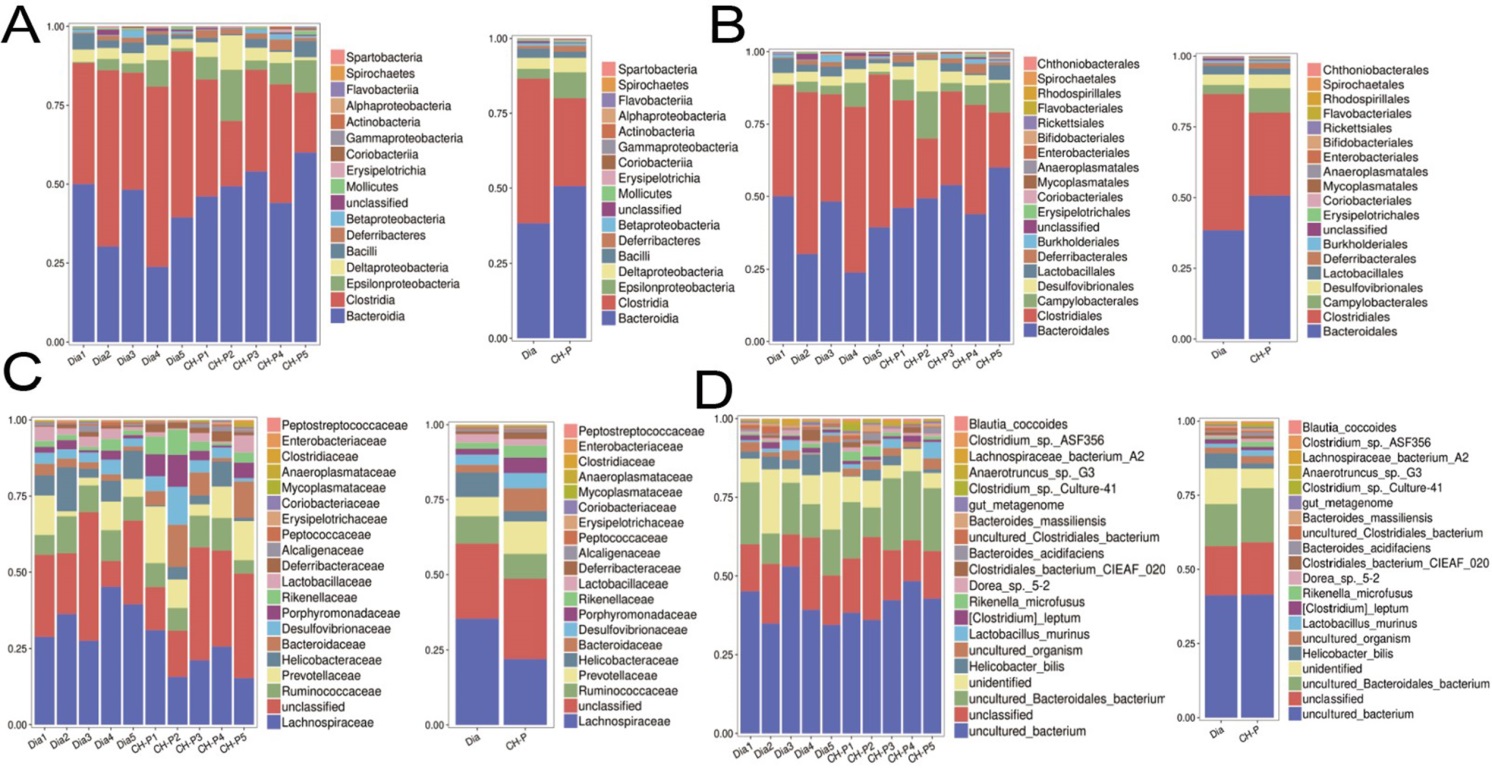


**Supplementary Figure 2.** Gut microbiome diversity and composition analysis. (A) Relative abundance of gut microbiota at the class level. (B) Relative abundance of gut microbiota at the order level. (C) Relative abundance of gut microbiota at the family level. (D) Relative abundance of gut microbiota at the species level.


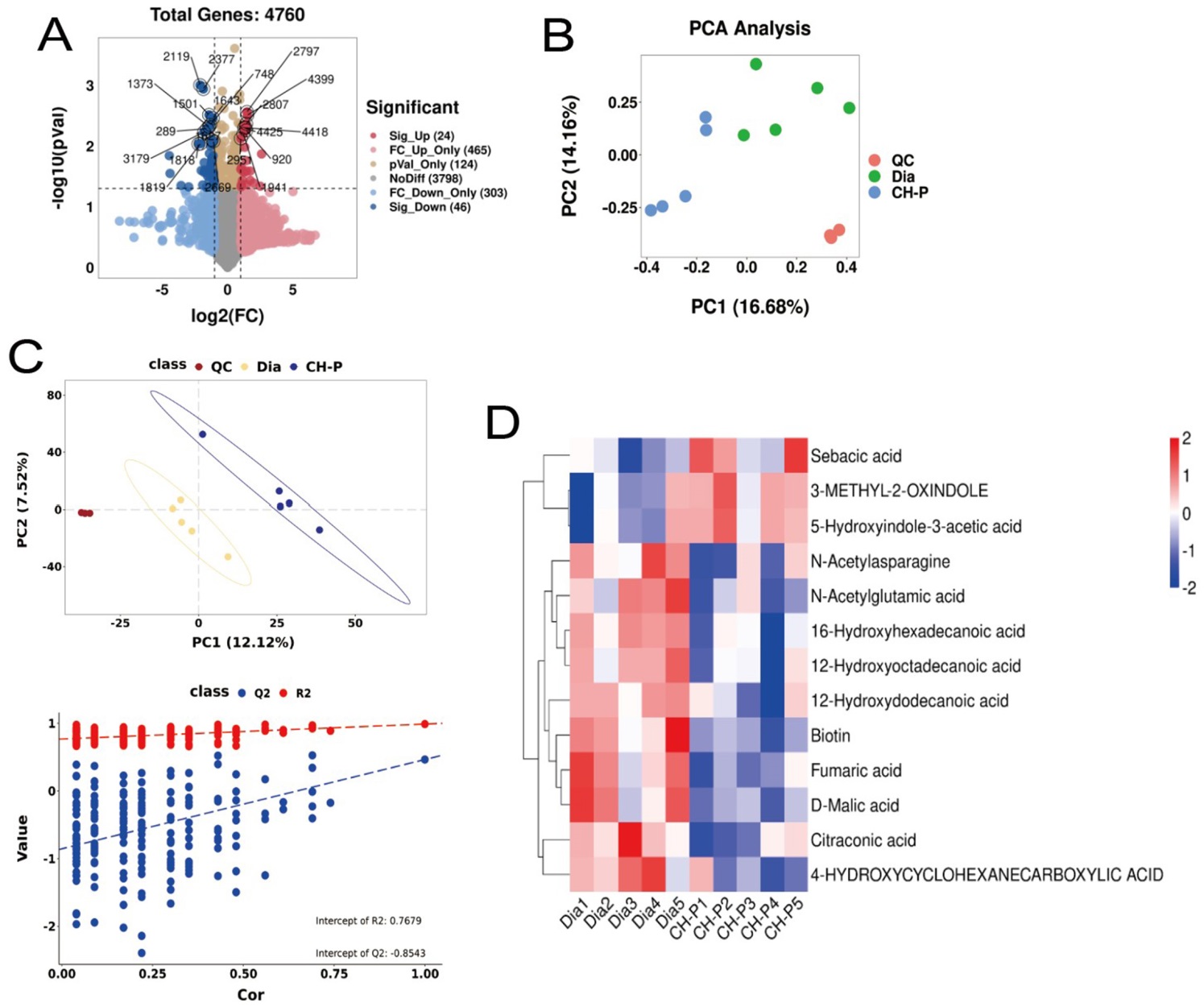


**Supplementary Figure 3.** Untargeted metabolomics analysis of the metabolites in the diabetic and CH-P treatment groups using the NEG model. (A) Principal component analysis score plot showing comparisons of the metabolomic profiles in both groups. (B) Volcano plot showing the differentially accumulated and significantly changed metabolites. (C) The PLS-DA score plot comparing the metabolome profiles in both groups and statistical validation of the PLS-DA model by permutation testing. (D) Hierarchical clustering heatmap of metabolites showing metabolites that significantly differ in abundance between both groups.


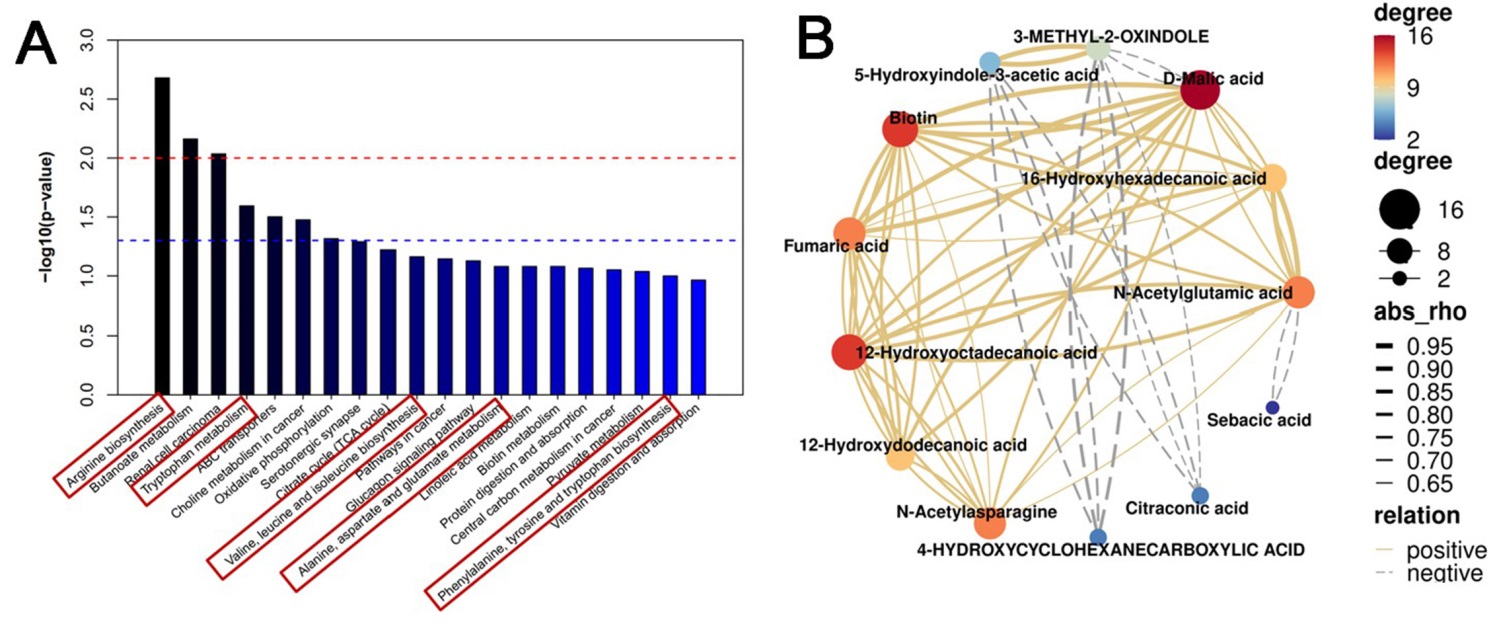


**Supplementary Figure 4.** Metabolic pathways involving the differential metabolites and their correlation. (A) Pathway enrichment analysis of the differential metabolites. (B) Correlation analysis of the differential metabolites using the NEG model.
